# MTA3-SOX2 Module Regulates Cancer Stemness and Contributes to Clinical Outcomes of Tongue Carcinoma

**DOI:** 10.3389/fonc.2019.00816

**Published:** 2019-08-27

**Authors:** Zhimeng Yao, Liang Du, Min Xu, Kai Li, Haipeng Guo, Guodong Ye, Dianzheng Zhang, Robert P. Coppes, Hao Zhang

**Affiliations:** ^1^Cancer Research Center, Shantou University Medical College, Shantou, China; ^2^Department of Biomedical Sciences of Cells and Systems, Section Molecular Cell Biology and Radiation Oncology, University Medical Center Groningen, University of Groningen, Groningen, Netherlands; ^3^Department of Head and Neck Surgery, Cancer Hospital of Shantou University Medical College, Shantou, China; ^4^Institute of Precision Cancer Medicine and Pathology, Jinan University Medical College, Guangzhou, China; ^5^Department of Bio-Medical Sciences, Philadelphia College of Osteopathic Medicine, Philadelphia, PA, United States; ^6^Research Centre of Translational Medicine, The Second Affiliated Hospital of Shantou University Medical College, Shantou, China

**Keywords:** tongue squamous cell carcinoma, MTA3, SOX2, cancer stem cell, prognosis, proliferation, progression

## Abstract

Cancer cell plasticity plays critical roles in both tumorigenesis and tumor progression. Metastasis-associated protein 3 (MTA3), a component of the nucleosome remodeling and histone deacetylase (NuRD) complex and multi-effect coregulator, can serve as a tumor suppressor in many cancer types. However, the role of MTA3 in tongue squamous cell cancer (TSCC) remains unclear although it is the most prevalent head and neck cancer and often with poor prognosis. By analyzing both published datasets and clinical specimens, we found that the level of MTA3 was lower in TSCC compared to normal tongue tissues. Data from gene set enrichment analysis (GSEA) also indicated that MTA3 was inversely correlated with cancer stemness. In addition, the levels of MTA3 in both samples from TSCC patients and TSCC cell lines were negatively correlated with SOX2, a key regulator of the plasticity of cancer stem cells (CSCs). We also found that SOX2 played an indispensable role in MTA3-mediated CSC repression. Using the mouse model mimicking human TSCC we demonstrated that the levels of MTA3 and SOX2 decreased and increased, respectively, during the process of tumorigenesis and progression. Finally, we showed that the patients in the MTA3^low^/SOX2^high^ group had the worst prognosis suggesting that MTA3^low^/SOX2^high^ can serve as an independent prognostic factor for TSCC patients. Altogether, our data suggest that MTA3 is capable of repressing TSCC CSC properties and tumor growth through downregulating SOX2 and MTA3^low^/SOX2^high^ might be a potential prognostic factor for TSCC patients.

## Introduction

More than 500,000 new cases of oral and pharyngeal cancers are diagnosed yearly worldwide (World Cancer report 2014, https://www.who.int/cancer/publications/WRC_2014/en/). Oral cancer is malignant neoplasia which arises on the lip or oral cavity. Although progress has been made in cancer treatments, the oral cancer survival rate has not been improved significantly for decades (1, 2). Since ~90% of these cancers are histologically shown to be originated from squamous cells (3), this subtype of cancers is traditionally defined as oral squamous cell carcinoma (OSCC). OSCC may show different levels of differentiation and has a propensity to coincide with lymph node metastasis (4). Among OSCC, tongue squamous cell cancer (TSCC) has the highest incidence and is usually associated with a poor survival rate. Therefore, TSCC is one of the most lethal types of cancers in the head and neck region (5). Thus, a better understanding of the underlying mechanisms in TSCC development will provide not only a more reliable biomarkers for diagnosis and prognosis but also potential therapeutic targets for the treatment of this cancer.

Cellular plasticity plays critical roles in tumor initiation, progression, and metastasis. It is now well-established that stem cell-like cancer cells or cancer stem cells (CSCs) are responsible for both cell plasticity and treatment (6–10). CSCs are a small subset of cancer cells and multiple lines of evidence indicate that CSCs are responsible for tumor initiation, indefinite, and progression (7, 11, 12). Accumulating data also indicate that plasticity of CSCs closely correlates with recurrences and metastasis (13–16) with poor prognosis in a wide variety of cancers, including tongue cancer (17–20).

Metastasis-associated protein 3 (MTA3) is a multi-effect coregulatory factor and plays indispensable roles in cell proliferation, tumorigenesis, and metastasis (21–26). Compelling evidence suggests that MTA3 is a tumor suppressor in many cancer types (26–28) by serving as an integral subunit of the nucleosome remodeling and histone deacetylase (NuRD) complex (21, 25, 27). As a transcriptional corepressor (29), MTA3 either directly or indirectly regulates the expression and activity of EMT-associated genes such as Snail and E-cadherin (25, 27). Dysregulation of MTA3 has been observed in many different human tumors (26–28). Reduced levels of MTA3 lead to the upregulation of Snail and subsequently enhance the process of epithelial-mesenchymal transition (EMT) (25, 27, 30). Consistently, the dysfunctional MTA3 reduces cell-cell adhesion and promotes cancer invasion and metastasis (23, 26). Moreover, reduced expression of MTA3 in tumor specimens has been associated with poor survival and therefore the expression of MTA3 has been suggested as an independent predictor of patient prognosis in uterine non-endometrioid carcinomas, gastroesophageal junction adenocarcinoma, glioma, and colorectal cancer (27, 28, 31, 32). However, the role of MTA3 and the underlying mechanism of MTA3's function in TSCC remain largely unknown.

In this study, we found that reduced levels of MTA3 in the patient specimens correlated with poorer clinical outcomes with concurrently increased cancer stemness. We also showed that MTA3 was capable of repressing cancer cell proliferation through inhibiting SOX2 expression. Using a chemical-induced mouse model of TSCC, we demonstrated that MTA3 and SOX2 decreased and increased, respectively, during the process of carcinogenesis and progression. Finally, our findings suggested that MTA3^low^/SOX2^high^ could potentially serve as an independent prognostic factor for TSCC patients.

## Materials and Methods

### Patient Tissue Samples

A total of 119 patients with TSCC were recruited at the Affiliated Tumor Hospital of Shantou University Medical College from 2009 to 2011 and their TSCC were clinically diagnosed and histologically confirmed. The primary TSCC specimens and their matched non-cancerous tissues were paraffin-embedded. Samples from patients who underwent preoperative radiotherapy or chemotherapy for TSCC were excluded. Clinical research protocols of this study were reviewed and approved by the Ethics Committee of Shantou University Medical College.

### Immunohistochemistry (IHC)

Tissue sections (4 μm) from the formalin-fixed paraffin-embedded clinical specimens or 4NQO-induced tongue tumor tissues were processed and immune-stained with antibodies against MTA3 (Catalog No. A300-160A, Bethyl, 1: 600), SOX2 (Catalog No. 23064, Cell Signaling, 1: 200), each with at least two cores of the primary tumor as well as two cores of normal tongue tissue. Sections immune-labeled with rabbit IgG or mouse IgG as the primary antibody were used as negative controls, known MTA3 and SOX2 positive slides were used as a positive control.

### IHC Evaluation

The percentage of positively stained cells were scored using the following scales: 0, no staining in any field; 1, ≤ 10; 2, 11–50; 3, 51–75; 4, > 75%. The intensity of staining was scored using the following scales: 1+, weak staining; 2+, moderate staining; 3+, strong staining. Percentage (P) and intensity (I) of nuclear, cytoplasm or membrane expression were multiplied to generate a numerical score (S = P • I).

The tissue sections were scored by two pathologists blind to the clinical outcomes. Receiver operating characteristic (ROC) curves were employed to define an optical cut-off score, which was closest to the point with maximum sensitivity and specificity. The cases with scores lower than or equal to the cut-off value were designated as low expression group and those with higher scores were categorized as high expression group.

### Histological Analysis

For histological analysis, tissues were fixed in 4% neutral buffer formalin, embedded in paraffin, sectioned (4 μm) and stained with hematoxylin and eosin.

### Gene Set Enrichment Analyses

Microarray data (accession no. GSE78060) were obtained from the Gene Expression Omnibus of NCBI (http://www.ncbi.nlm.nih.gov/geo/) and subjected to Gene set enrichment analysis (GSEA) using GSEA software (version 2.0.13) (http://www.broadinstitute.org/gsea/index.jsp).

### Immunofluorescence Staining

FFPE tissue sections were deparaffinized and dehydrated in xylene and graded ethanol solutions in preparation for MTA3 and SOX2 double immunofluorescence (IF) staining. All slides were subjected to heat-induced epitope retrieval in Citrate Buffer [pH = 6.0]. Endogenous tissue peroxidases were blocked by incubating the slides in 3% hydrogen peroxide solution and blocking buffer before incubation with MTA3 (Catalog A300-160A, Bethyl, 1: 600), SOX2 (Catalog No. #23064, Cell Signaling, 1: 200) as primary antibodies. And HRP-conjugated streptavidin as the secondary antibody. The signal in IF labeled slides were visualized with AlexaFluor 488 and AlexaFluor 594 Tyramide Super Boost kits (Invitrogen, Carlsbad, CA), and nuclei were visualized with Prolong Diamond Antifade Reagent with 4',6-diamidino-2-phenylindole (DAPI; Invitrogen, Carlsbad, CA). Primary and secondary antibodies were stripped using Citrate Buffer [pH = 6.0] in the microwave. Known MTA3 and SOX2 positive slides were used as a positive control. Immunofluorescence staining was analyzed using the PerkinElmer Vectra analysis platform to estimate the cell numbers. The percentage of positive cells was estimated by two pathologists.

### Cell Culture

Human TSCC cell lines (SCC-25 and SCC-4) were obtained from the Cell Bank of the Chinese Academy of Sciences (Shanghai, China). The cells were cultured in Dulbecco's Modified Eagle Media (DMEM, Gibco/Invitrogen) supplemented with 10% FBS (Gibco/Invitrogen) at 37°C in a humidified atmosphere containing 5% CO_2_.

### Virus Production and Transduction

The full-length cDNA of MTA3 was PCR amplified from SCC-25 cells and cloned into the pCDNA3.1-flag plasmid. The shRNA targeting human MTA3 (target sequence: GAGGATACCTTCTTCTACTCA) was cloned into pBabe/U6 plasmid. The SOX2 overexpression plasmid and SOX2 short hairpin RNA (shSOX2) plasmid (shSOX2 target sequence: GGTTGACACCGTTGGTAATTT) were obtained from GeneCopoeia. Transfection of plasmid was performed using Lipofectamine 3000 (Thermo Fisher Scientific, catalog no. L3000015) according to the manufacturer's instructions. Stable cells were selected by culturing the cells in the medial with puromycin for 2 weeks.

### RNA Isolation and Quantitative Real-Time PCR

Total RNA was extracted from cells using TRIzol (Invitrogen) according to the manufacturer's instruction and 2 μg RNA was reversely transcribed using High Capacity cDNA Reverse Transcription Kit (Applied Biosystems, Foster City, CA, USA). The cDNA was amplified and quantified in ABI-7500 system (Applied Biosystems) using SYBR Green Master (Roche). The cDNA was subjected to quantitative real-time PCR (qPCR) with the following primers: MTA3 forward: 5′-AAGCCTGGTGCTGTGAAT-3′ and reverse: 5′-AGGGTCCTCTGTAGTTGG-3′; SOX2 forward: 5′-CATCACCCACAGCAAATGACA-3′ and reverse: 5′-GCTCCTACCGTACCACTAGAACTT-3′; GADPH forward: 5′-TCCTCCTGTTTCATCCAAGC-3′ and reverse: 5′-TAGTAGCCGGGCCCTACTTT-3′.

### Western Blot Analysis

Whole-cell lysates were prepared by lysing the cells in lysis buffer. Cell lysates with an equal amount of proteins were separated on 10% SDS-PAGE and transferred to PVDF membranes. The membranes were incubated with primary antibodies (MTA3, Catalog No. A300-160A, Bethyl, 1: 2,000; SOX2, Catalog No.23064, Cell Signaling, 1: 1,000; GAPDH, Catalog No. ab8245, Abcam, 1:3,000) followed by HRP-conjugated secondary antibodies as previously described (33). Blotted proteins were visualized by incubating in SuperSignal West Pico Chemiluminescent Substrate (Thermo Scientific) followed by exposure to X-ray film (Eastman Kodak, Rochester, NY, USA) (33).

### ALDEFLUOR Assay

ALDEFLUOR assay kit (Stem Cell Technologies^TM^, Vancouver, BC, Canada) was used to determine ALDH1 activity according to the manufacturer's protocol. Cells were suspended in ALDEFLUOR assay buffer containing 1 μM per 1 × 10^6^ cells of the ALDH substrate, boron-dipyrromethene-aminoacetaldehyde (BAAA), and incubated for 50 min at 37°C. Each sample was treated with 50 mM of an ALDH-specific inhibitor, and diethylaminobenzaldehyde (DEAB) as a negative control. Stained cells were analyzed by BD FACSAria^TM^ II (BD Biosciences, San Jose, CA, USA). To evaluate cell viability the cells were stained with 1 mg/ml of propidium iodide prior to analysis.

### Proliferation and Survival Assays

Real-time cell analysis (RTCA) was performed to estimate cell proliferation using the xCELLigence DP device (ACEA Biosciences) as described in the supplier's instructions. In brief, 3,000 cells were seeded in E-plates, and the plates were locked into the RTCA DP device supplied with humidified air with 5% CO_2_ at 37°C. The proliferative ability was monitored by the xCELLigence RTCA Analyzer (Roche Applied Science, Mannheim, Germany) (34, 35).

### Animals and Carcinogen Treatment

Male and female wild-type C57BL/six mice were supplied by Beijing Vital Laboratory Animal Technology (Beijing, China). To induce tumorigenesis in the tongue, 4NQO (Sigma-Aldrich, St. Louise, MO) was added to the drinking water (100 mg/mL) for the 6-week-old young adult mice for 16 weeks. The mice were sacrificed when the bodyweight loss >1/3, otherwise they were sacrificed at the indicated time. After sacrifice, the tongue surface was photographed. The tissues were resected for histopathological examination and immunohistochemistry (IHC) analyses. Animals were housed in pathogen-free conditions at the Animal Center of Shantou University Medical College in compliance with Institutional Animal Care and Use Committee (IACUC) regulations (SUMC2014-148). All animal experiments were performed according to protocols approved by the Animal Care and Use Committee of the Medical College of Shantou University.

### Gaussia Luciferase Assay

The pEZX-PG04 plasmid carrying double-expression cassette for *Gaussia* luciferase under the control of the SOX2 promoter (+270 to −1038), and secreted Alkaline Phosphatase (SeAP) under the control of the CMV promoter was obtained from GeneCopoeia (Catalog No. HPRM15202). Cells were seeded in 24-well plates, and transiently transfected with the above plasmid using Lipofectamine 3000 (Thermo Fisher Scientific, catalog no. L3000015) according to the manufacturer's instructions. After 72 h of transfection, the culture medium was collected for analysis of *Gaussia* luciferase and secreted Alkaline Phosphatase (SeAP) activities using a Secrete-PairTM Dual Luminescence Assay Kit (GeneCopoeia, SPDA-D010) according to the manufacturer's instructions. *Gaussia* luciferase activity was normalized on the basis of seAP activity.

### Statistical Analyses

All statistical analyses except for microarray data were carried out using the statistical software package SPSS 17.0 (SPSS, Inc., Chicago, IL, USA). The comparisons between two groups were performed with Student's *t*-test. The correlation between MTA3 expression and clinicopathological data of patients was analyzed with Pearson χ2 test. Survival curves were plotted with the Kaplan-Meier method and compared by log-rank test. Survival data were evaluated by univariate and multivariate Cox regression analyses. The correlations of the histoscore between MTA3 and SOX2 was determined by Spearman's rank test. Two-way ANOVA followed by a Tukey–Kramer *post hoc* test was performed to compare the difference of proliferation affected by MTA3 and SOX2 among four groups. All data were presented as the mean ± SEM. The *P* < 0.05 was considered statistically significant.

## Results

### MTA3 Is Reduced in Human TSCC

**Figure 1:**
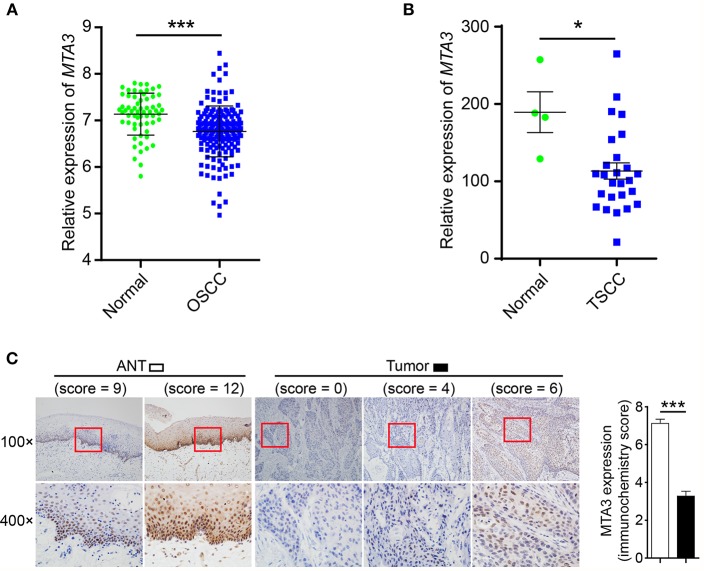
MTA3 is downregulated in human TSCC. **(A)** Analysis of *MTA3* mRNA expression was performed in an OSCC dataset from GEO (GSE30784). **(B)** *MTA3* mRNA expression was analyzed in a TSCC dataset from GEO (GSE78060). **(C)** MTA3 expression in 119 human TSCC tissues and paired adjacent normal tissues (ANT) was monitored by immunohistochemistry (IHC) (left panel). The immunohistochemistry score of MTA3 in TSCC (filled bar) and paired normal adjacent (open bar) tissues were plotted (right panel). Shown are the mean values or representative data from at least three independent experiments. Error bars indicate SEM. *P < 0.05, ***P < 0.001 using student's *t*-test.

To estimate the expression MTA3, we first assessed the mRNA levels of MTA3 in OSCC from GEO database (https://www.ncbi.nlm.nih.gov/geo/) GSE30784 (36) and GSE25099 (37). We found that the MTA3 mRNA levels were significantly lower in OSCC when compared with the normal controls (*P* < 0.001 and 0.01, respectively; Figure 1A and Supplementary Figure 1A). Since TSCC is the highest incidence of all oral squamous cell cancers (5), we focused on the role of MTA3 in TSCC. Data from both datasets GSE78060 (38) and GSE34105 (39) revealed higher *MTA3* mRNA levels in normal tongue tissues than in TSCC tissues (*P* = 0.014 and 0.003, respectively; Figure 1B and Supplementary Figure 1B). Next, we examined the MTA3 expression at protein levels in TSCC of 119 patient specimens using immunohistochemistry (IHC). Representative photomicrographs for MTA3 IHC scores of level 0, 4, 6, 9, and 12 are shown in Figure 1C (left panel). TSCC showed significantly (*P* < 0.001, *n* = 119) lower levels of MTA3 protein in the primary tumors compared to the corresponding normal tissue (Figure 1C, Right panel). These findings demonstrate that MTA3 is downregulated in TSCC tissues compared to normal controls.

### Downregulation of MTA3 Correlates With Clinical Outcomes in TSCC Patients

**Figure 2:**
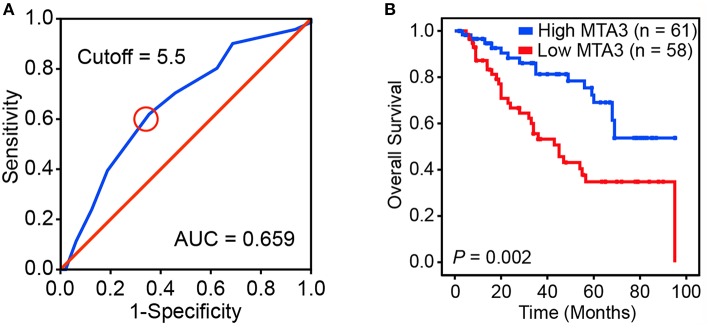
Downregulation of MTA3 correlates with poor prognosis in human TSCC. **(A)** Receiver operating characteristic (ROC) curve analysis was performed to determine the cut-off score for the low expression of MTA3. **(B)** Kaplan–Meier curves compared the overall survival in TSCC patients with high and low protein levels of MTA3.

We next assessed the prognostic impact of MTA3 on the outcome of TSCC patients. An optimal cutoff value was identified using Receiver operator characteristic (ROC) analysis which categorized 51.3% (61/119) of the patient cohort into a high MTA3 group and the remainder into a low MTA3 group (Figure 2A). Then Kaplan-Meier survival analyses were performed and showed that patients with low MTA3 were associated with shorter overall survival than those with high MTA3 (*P* = 0.002, Figure 2B).

Univariate analyses found that MTA3 expression, pTNM stage, pN status, and tumor depth were significantly related to TSCC patient outcome (Table 1). However, after multivariate Cox regression analysis only MTA3 expression (HR 0.420; 95% CI 0.218–0.810; *P* = 0.010) and pTNM stage (HR 3.029; 95% CI 1.075–8.538; *P* = 0.036) were independently significant with overall survival (Table 1). These results reveal that reduced expression of MTA3 may be an independent prognostic factor for the overall survival of patients with TSCC.

**Table 1:** Univariate and multivariate Cox proportional hazards analyses of survival in TSCC patients.

**Variables**	**Univariate analysis**	***P-*value**	**Multivariate analysis**	***P*-value**
	**HR (95% CI)**		**HR (95% CI)**	
Age (years)				
> 60 vs. ≤ 60	1.106 (0.620–1.973)	0.734	1.171 (0.633–2.167)	0.616
Gender				
Male vs. Female	1.620 (0.909–2.886)	0.102	1.630 (0.882–3.010)	0.119
Differentiation				
Poor vs. Well/Moderate	1.418 (0.684–2.936)	0.348	1.159 (0.533–2.518)	0.709
pTNM stage				
III–IV vs. I–II	4.428 (2.284–8.583)	0.000	3.029 (1.075–8.538)	0.036
MTA3 expression				
High vs. Low	0.401 (0.221–0.727)	0.003	0.420 (0.218–0.810)	0.010
pN status				
N1–N3 vs. N0	2.991 (1.672–5.349)	0.000	1.340 (0.590–3.047)	0.484
Tumor depth				
T1/T2 vs. T3/T4	2.472 (1.373–4.449)	0.003	1.064 (0.518–2.187)	0.865

HR, hazard ratio; CI, confidence interval.

*High in this analysis is based on an MTA3 level > 5.5; the remaining individuals were classified as low*.

### MTA3 Inhibits Key TSCC Plasticity Regulator SOX2

**Figure 3:**
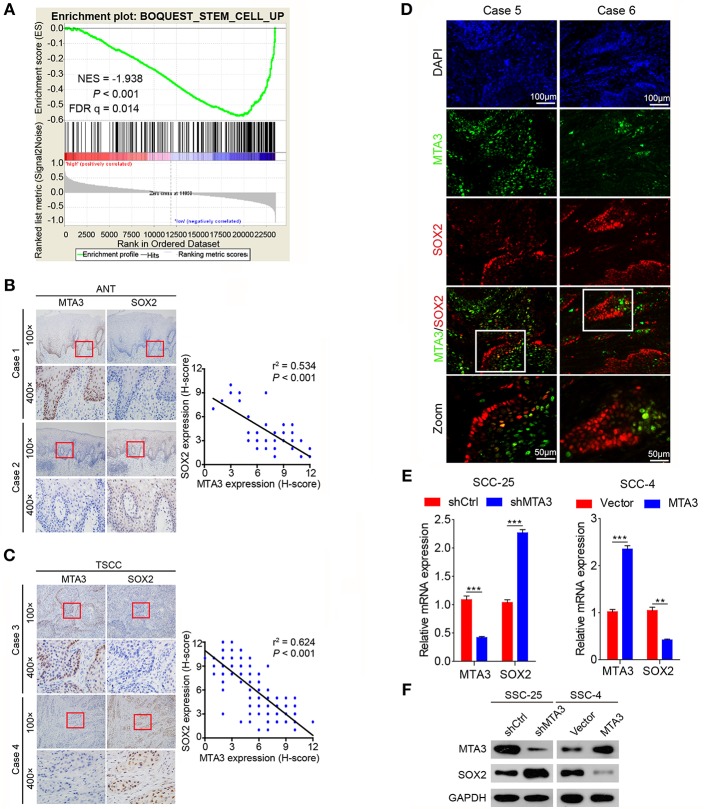
MTA3 represses key TSCC plasticity regulator SOX2. **(A)** GSEA plots of enrichment of BOQUEST_STEM_CELL_UP signatures in MTA3^High^ tumors vs. MTA3^Low^ tumors in the GSE78060 dataset. **(B)** Representative IHC image against MTA3 and SOX2 of normal tissues (left panel), Pearson's correlation analysis of MTA3 and SOX2 IHC intensity in 119 adjacent non-tumor tissues (ANT) (right panel). **(C)** Representative IHC image against MTA3 and SOX2 of TSCC tissues (left panel), Pearson's correlation analysis of MTA3 and SOX2 IHC intensity in 119 TSCC tissues (right panel). **(D)** Representative double immunofluorescence of MTA3 and SOX2 in TSCC patient tissues. DAPI = blue, MTA3 = green, SOX2 = red. **(E)** qRT-PCR of MTA3 and SOX2 in SCC-25 cells with MTA3 depletion (Left panel) and SCC-4 cells MTA3 overexpression (Right panel), **P < 0.01 and ***P < 0.001 using student's *t*-test. **(F)** Western blot of MTA3 and SOX2 in SCC-25 cells with MTA3 depletion (Left panel) and SCC-4 cells with MTA3 overexpression (Right panel).

The plasticity of CSC plays an important role in oncogenesis and progression (7, 11, 12, 40). Therefore, we next explored the relationship between MTA3 level and cancer cell stemness using gene set enrichment analysis (GSEA) of published human TSCC expression profiles (GSE78060) and found that a cancer stemness related gene signature (BOQUEST_STEM_CELL_UP) was significantly enriched in TSCC with low MTA3 expression (*P* < 0.001, Figure 3A). Interestingly, SOX2, a key regulator in the plasticity of cancer stemness, was also closely associated with poor prognosis in TSCC patients (20). To study the relation between MTA3 and SOX2, we measured the (co-)expression of MTA3 and SOX2 in adjacent non-tumor tissues (ANT) and TSCC tissues from the 119 patients. Indeed, a highly significant inverse correlation between the MTA3 and SOX2 levels (*r*^2^ = 0.534, *P* < 0.001 and *r*^2^ = 0.624, *P* < 0.001, respectively; Figures 3B,C) was observed. Moreover, a low level of SOX2 was accompanied by high expression of MTA3, and vice versa, as indicated by double immunofluorescent staining (Figure 3D). In addition, two TSCC cell lines, SSC-25 and SSC-4, were transfected with shMTA3 or the plasmid overexpressing MTA3, respectively. The mRNA and protein levels of MTA3 were then assessed by qRT-PCR and western blot analysis, respectively. When compared to the controls, the expression level of MTA3 was obviously enhanced in cells transfected with pcMTA3 (*P* < 0.001), while it was significantly decreased in cells transfected with shMTA3 (*P* < 0.001) (Figures 3E,F). Knockdown MTA3 dramatically increased, whereas overexpression of MTA3 decreased, the expression levels of SOX2 (Figures 3E,F), suggesting that the MTA3 inhibits SOX2 expression in TSCC cells.

### MTA3 Suppresses TSCC Cell Stemness and Proliferation Via SOX2

**Figure 4:**
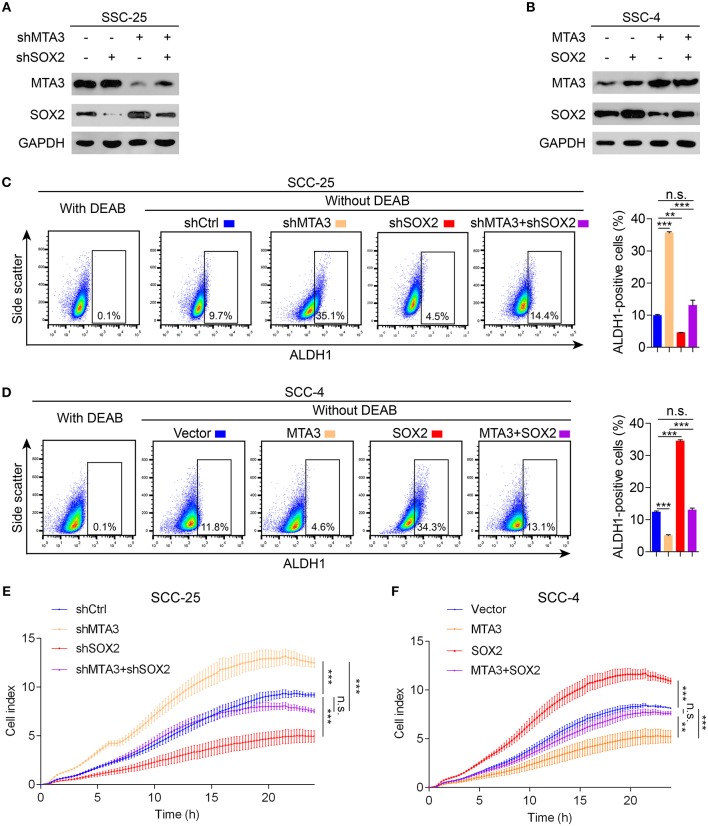
MTA3 reduces CSC properties and cell proliferation via SOX2 in TSCC cells. **(A,B)** Western blot and qRT-PCR in TSCC cells transfected with a combination of shMTA3 and shSOX2 **(A)** or MTA3 and SOX2 expressing plasmid **(B)**. **(C,D)** Flow cytometry analysis of the ALDH1^+^ population. Histograms showing the proportion of ALDH1^+^ cells. **(E,F)** Cells were monitored by RTCA for 24 h. Data were shown as the means of three independent experiments or representative data. Error bars indicate SEM. n.s., not statistically significant; **P < 0.01, ***P < 0.001 by two-way ANOVA followed by a Tukey–Kramer *post hoc* test.

To determine whether SOX2 plays a key role in MTA3-mediated plasticity of CSC and cell growth, we first established TSCC cells with knockdown of MTA3 or SOX2 alone or in combination using specific shRNA (Figure 4A) or stably overexpressing MTA3 or SOX2 alone or in combination (Figure 4B). As expected, knockdown MTA3 significantly promoted the percentage of cells expressing ALDH1, a major CSC marker (Figure 4C). MTA3-depletion-mediated increase of the number of ALDH1-positive cells were significantly reduced when SOX2 is removed suggesting SOX2 plays a crucial role in MTA3-repressed CSC properties (Figure 4C). Next, the effects of overexpressed MTA3 individually or in combination with SOX2 were examined. Figure 4D showed that overexpression of MTA3 and SOX2 reduced and increased the number of ALDH1-positive cells, respectively. However, overexpressed SOX2 counteracted MTA3-repressed the number of ALDH1-positive cells (Figure 4D). In addition, real-time proliferation assays were conducted to assess changes in cell dynamics. Silencing SOX2 inhibited MTA3-depletion-induced cell proliferation (Figure 4E) and forced SOX2 expression was capable of counteracting MTA3-repressed cell proliferation (Figure 4F). These data altogether demonstrate that in TSCC cells MTA3 represses CSC property and proliferation by targeting SOX2.

### Dysregulated MTA3/SOX2 Axis Is Associated With Tumor Progression

**Figure 5:**
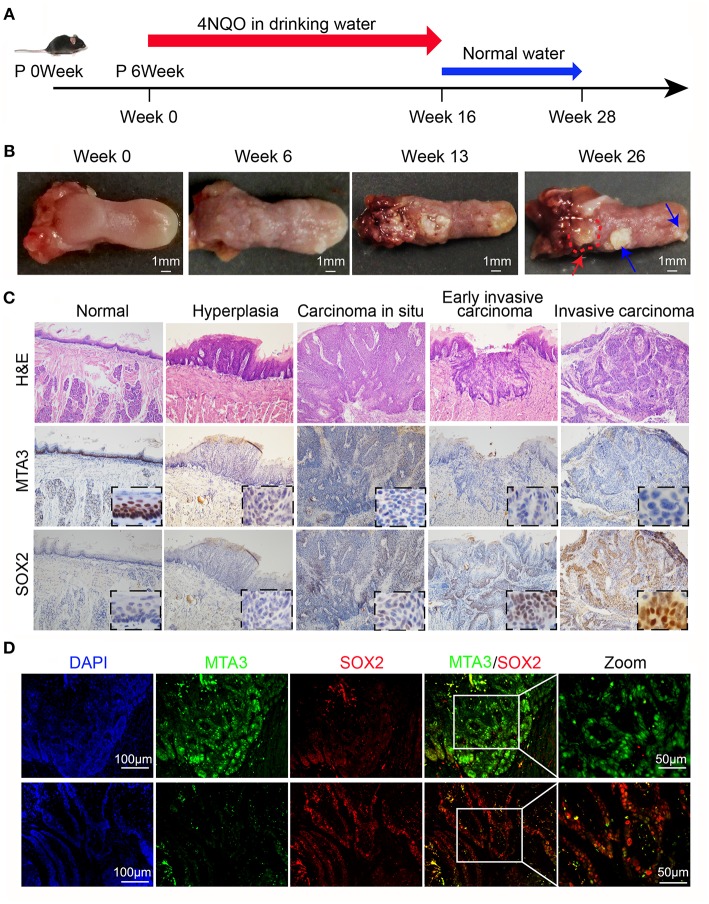
MTA3-SOX2 axis changes dynamically in a mouse model mimicking human TSCC. **(A)** Schematic representation of 4NQO tumorigenesis protocol in wild-type C57BL/6 mice. **(B)** Representative macroscopic view of the normal tongue and tumor development. The red arrow indicates invasive carcinoma, the blue arrow indicates papilloma. **(C)** Representative H&E and IHC stain for mice tongue preneoplastic and neoplastic tissues. **(D)** Representative double immunofluorescent staining image against MTA3 and SOX2 of mice tongue neoplastic tissues. DAPI = blue, MTA3 = green, SOX2 = red.

The results above indicate a role of MTA3/SOX2 in TSCC progression. Therefore, we next investigated the role of MTA3/SOX2 in a mouse model of tongue tumorigenesis induced by 4 nitroquinoline 1-oxide (4NQO). Exposure to 4NQO caused a temporal progression from hyperplasia to invasive carcinoma in the murine tongue, resembling human tongue carcinogenesis and development (41, 42). Mice were exposed to 4NQO in daily drinking water for 16 weeks followed by 4NQO-free drinking water for 12 additional weeks (Figure 5A), which resulted in tongue carcinogenesis and progression (Figure 5B) similar to what has been published previously (41, 42). Next, we studied the expression of MTA3/SOX2 in normal tongue tissue samples, hyperplasia, carcinoma *in situ*, early invasive carcinoma and invasive carcinoma by immunohistochemistry. As shown in Figure 5C, the expression of MTA3 gradually decreased during the process of carcinogenesis and progression, in contrast to SOX2. This is indicative of an inverse relation between MTA3 and SOX2 associated with cancer occurrence and development, which was confirmed in mouse TSCC by double immunofluorescent staining (Figure 5D). Thus, the levels of MTA3 and SOX2 may change dynamically during tongue carcinogenesis and progression.

### Dysregulated MTA3/SOX2 Axis Associated Strongly With Poor Prognosis

**Figure 6:**
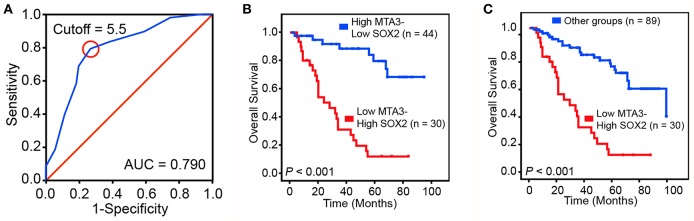
Dysregulated MTA3-SOX2 axis is associated with poor prognosis. **(A)** Receiver operating characteristic (ROC) curve analysis was performed to determine the cut-off score for the low expression of SOX2. **(B,C)** The overall survival of TSCC patients with tumors expressing low levels of MTA3 and high levels of SOX2 and those with high levels of MTA3 and low levels of SOX2 **(B)**, as well as all other subjects **(C)**.

To validate our findings in a clinical setting, we assessed the levels of MTA3 and SOX2 in 119 TSCC samples and related this to the overall survival rate. Receiver operator characteristic (ROC) analysis identified an optimal cutoff value and categorized 39.5% (47/119) of the patients into a high SOX2 group and the remainder into a low SOX2 group (Figure 6A). Indeed, patients with low levels of MTA3 but high levels of SOX2 (MTA3^low^/SOX2^high^) had significantly shorter overall survival rate (*P* < 0.001; Figure 6B) than those with high levels of MTA3. Moreover, low levels of SOX2 (MTA3^high^/SOX2^low^), were associated with an even shorter overall survival compared to TSCC patients with any other expression pattern (MTA3^high^/SOX2^high^, MTA3^low^/SOX2^high^, MTA3^low^/SOX2^low^) (*P* < 0.001; Figure 6C). These data altogether suggest that combined MTA3^low^-SOX2^high^ expression had stronger correlation with worst patient prognosis than that of the individual components. Indeed, univariate and multivariate Cox regression analysis (*P* < 0.001 and *P* < 0.001, HR = 4.044, 95 %CI = 1.925–8.495, respectively) indicated that combined MTA3^low^/SOX2^high^ expression is an independent prognostic factor of TSCC as it was significantly associated with prognosis (Table 2). Overall, these data indicate that dysregulated MTA3/SOX2 axis may contribute to patient outcomes and could be of value as a predictive biomarker for TSCC prognosis.

**Table 2:** Univariate and multivariate Cox proportional hazards model predicting survival in TSCC patients.

**Variables**	**Univariate analysis**	***P-*value**	**Multivariate analysis**	***P*-value**
	**HR (95% CI)**		**HR (95% CI)**	
Age (years)				
> 60 vs. ≤ 60	1.106 (0.620–1.973)	0.734	1.310 (0.714–2.404)	0.383
Gender				
Male vs. Female	1.620 (0.909–2.886)	0.102	1.623 (0.878–3.000)	0.123
Differentiation				
Poor vs. Well/Moderate	1.418 (0.684–2.936)	0.348	1.211 (0.581–2.525)	0.610
pTNM stage				
III–IV vs. I–II	4.428 (2.284–8.583)	0.000	1.642 (0.569–4.735)	0.359
Combination of MTA3 and SOX2				
MTA3 Low/SOX2 High vs. Other groups	5.209 (2.891–9.386)	0.000	4.044 (1.925–8.495)	0.000
pN status				
N1–N3 vs. N0	2.991 (1.672–5.349)	0.000	1.495 (0.673–3.318)	0.323
Tumor depth				
T1/T2 vs. T3/T4	2.472 (1.373–4.449)	0.003	1.069 (0.533–2.142)	0.852

*HR, hazard ratio; CI, confidence interval*.

## Discussion

Here we demonstrated that MTA3 was a potential independent prognosis factor for TSCC patients. Moreover, we established the negative regulation of SOX2, a key regulator in the plasticity of cancer stemness, by MTA3 repressed the number of ALDH1-positive cells and cell proliferation in TSCC cells. We identified the expression of MTA3 and SOX2 as important markers in a 4NQO-induced TSCC mouse model, human TSCC progression and clinical outcomes.

The biological and clinical significance of the MTA family members in malignancies has been well-established. The levels of these factors have even been proposed as potential diagnostic parameters and targeting each one of the family members could be potential treatments for different cancers (26, 43–45). However, unlike MTA1 and MTA2 which were mainly involved in cancer progression and metastasis, MTA3 possesses both tumor-suppressing and tumor-promoting properties depending on specific cancer types (21, 43). We found that MTA3 was negatively associated with overall survival and could act as an independent prognosis factor in TSCC. To the best of our knowledge, this is the first study to show the expression of MTA3 in TSCC.

The plasticity of CSCs is key mechanisms in oncogenesis and progression of cancers (7, 11, 12, 16) and a predictive factor of poor prognosis in a wide variety of cancers, including tongue cancer (17–20). Accumulating evidence suggests that transcription factor SOX2 is a key regulator in the plasticity of cancer stemness (46–48). Moreover, overexpression of SOX2 is often associated with increased cancer aggressiveness, resistance to chemoradiation therapy and decreased survival rate, which has been reported in various cancer types (49, 50), including TSCC (51). Previous studies revealed that SOX2 is vital in the regulation of TSCC motility, invasion, tumorigenicity, and upregulated SOX2 is significantly associated with the progression of TSCC (51). SOX2 is detectable in oral pre-invasive lesions, suggesting that SOX2 upregulation may be an early event in TSCC carcinogenesis. We found that MTA3 can inhibit CSC properties and cell proliferation via downregulating SOX2 in TSCC cells. In addition, luciferase reporter assays showed that knockdown or overexpression MTA3 had no significant effect on the luciferase activity (Supplementary Figure 2), suggesting that MTA3 does not regulate the expression of SOX2 by interacting with its proximal promoter. However, these results do not necessarily rule out the possibility that MTA3 is directly involved in the regulation of SOX2 expression by interacting with its enhancer region or the sequence other than the proximal promoter. Further studies will better the understanding about the mechanisms in MTA3-regulated SOX2 expression in tongue cancer.

Given the fact that alterations of MTA3 and SOX2 highly correlate with clinical outcomes and the predictability of prognosis, the levels of MTA3 and SOX2, especially MTA3^low^/SOX2^high^, could be used as diagnostic parameters. In addition, targeting MTA3 and/or SOX2 could be potential therapeutic strategies in TSCC treatment. Finally, 4NQO-induce TSCC mice model has been widely used in the study of TSCC (41, 42). And we used this model to further elaborate the dynamic changes of MTA3 and SOX2 in the occurrence and developmental progression of TSCC.

In summary, consistent with the fact that MTA3 is often silenced in the process of carcinogenesis and development, we found that MTA3 was capable of inhibiting CSC properties and cell proliferation by negatively regulating SOX2. Additionally, we found that TSCC patients in the MTA3^low^/SOX2^high^ group had a poor prognosis. Future research may involve translational research to clinically evaluate the efficacy of this therapeutic strategy using IHC evaluation of low MTA3 and high SOX2 as a companion diagnostic for patient selection.

## Data Availability

The datasets generated for this study are available on request to the corresponding author.

## Author Contributions

HZ designed the experiments. ZY and LD performed the *in vivo* experiments. ZY, KL, and LD assayed and analyzed of patient samples. LD and KL performed bioinformatics analyses. LD, MX, KL, ZY, and GY performed the *in vitro* experiments. LD, MX, KL, and ZY performed the statistical analysis. MX and HG collected the clinical samples. DZ and HZ analyzed data. HZ, LD, RC, and DZ prepared the manuscript. All the authors read and approved the final manuscript.

### Conflict of Interest Statement

The authors declare that the research was conducted in the absence of any commercial or financial relationships that could be construed as a potential conflict of interest.

## References

[1] Rivera C. Essentials of oral cancer. Int J Clin Exp Pathol. (2015) 8:11884–94. 10.5281/zenodo.192487 26617944 PMC4637760

[2] Kademani D. Oral cancer. Mayo Clin Proc. (2007) 82:878–87. 10.4065/82.7.878 17605971

[3] Lingen MW Kalmar JR Karrison T Speight PM. Critical evaluation of diagnostic aids for the detection of oral cancer. Oral Oncol. (2008) 44:10–22. 10.1016/j.oraloncology.2007.06.011 17825602 PMC2424250

[4] Ho AS Kim S Tighiouart M Gudino C Mita A Scher KS. Metastatic lymph node burden and survival in oral cavity cancer. J Clin Oncol. (2017) 35:3601–9. 10.1200/JCO.2016.71.1176 28880746 PMC5791830

[5] Ganly I Patel S Shah J. Early stage squamous cell cancer of the oral tongue–clinicopathologic features affecting outcome. Cancer. (2012) 118:101–11. 10.1002/cncr.26229 21717431

[6] Carnero A Garcia-Mayea Y Mir C Lorente J Rubio IT Me LL. The cancer stem-cell signaling network and resistance to therapy. Cancer Treat Rev. (2016) 49:25–36. 10.1016/j.ctrv.2016.07.001 27434881

[7] Lytle NK Barber AG Reya T Stem cell fate in cancer growth, progression and therapy resistance. Nature reviews. Cancer. (2018) 18:669–80. 10.1038/s41568-018-0056-x PMC8388042 30228301

[8] Cojoc M Mabert K Muders MH Dubrovska A. A role for cancer stem cells in therapy resistance: cellular and molecular mechanisms. Semin Cancer Biol. (2015) 31:16–27. 10.1016/j.semcancer.2014.06.004 24956577

[9] Batlle E Clevers H. Cancer stem cells revisited. Nat Med. (2017) 23:1124–34. 10.1038/nm.4409 28985214

[10] Vlashi E Pajonk F. Cancer stem cells, cancer cell plasticity and radiation therapy. Semin Cancer Biol. (2015) 31:28–35. 10.1016/j.semcancer.2014.07.001 25025713 PMC4291301

[11] Hayashi H Higashi T Yokoyama N Kaida T Sakamoto K Fukushima Y. An imbalance in TAZ and YAP expression in hepatocellular carcinoma confers cancer stem Cell-like behaviors contributing to disease progression. Cancer Res. (2015) 75:4985–97. 10.1158/0008-5472.CAN-15-0291 26420216

[12] Li C Wang J. Quantifying the landscape for development and cancer from a core cancer stem cell circuit. Cancer Res. (2015) 75:2607–18. 10.1158/0008-5472.CAN-15-0079 25972342

[13] de Sousa e Melo F Kurtova AV Harnoss JM Kljavin N Hoeck JD Hung J. A distinct role for Lgr5(+) stem cells in primary and metastatic colon cancer. Nature. (2017) 543:676–80. 10.1038/nature21713 28358093

[14] Samanta D Park Y Andrabi SA Shelton LM Gilkes DM Semenza GL. PHGDH expression is required for mitochondrial redox homeostasis, breast cancer stem cell maintenance, and lung metastasis. Cancer Res. (2016) 76:4430–2. 10.1158/0008-5472.CAN-16-0530 27280394

[15] Lawson DA Bhakta NR Kessenbrock K Prummel KD Yu Y Takai K. Single-cell analysis reveals a stem-cell program in human metastatic breast cancer cells. Nature. (2015) 526:131–5. 10.1038/nature15260 26416748 PMC4648562

[16] Li Y Rogoff HA Keates S Gao Y Murikipudi S Mikule K. Suppression of cancer relapse and metastasis by inhibiting cancer stemness. Proc Natl Acad Sci USA. (2015) 112:1839–44. 10.1073/pnas.1424171112 25605917 PMC4330785

[17] de Sousa EMF Colak S Buikhuisen J Koster J Cameron K de Jong JH Methylation of cancer-stem-cell-associated Wnt target genes predicts poor prognosis in colorectal cancer patients. Cell Stem Cell. (2011) 9:476–85. 10.1016/j.stem.2011.10.008 22056143

[18] Zheng H Pomyen Y Hernandez MO Li C Livak F Tang W. Single-cell analysis reveals cancer stem cell heterogeneity in hepatocellular carcinoma. Hepatology. (2018) 68:127–40. 10.1002/hep.29778 29315726 PMC6033650

[19] Peng F Wang JH Fan WJ Meng YT Li MM Li TT Glycolysis gatekeeper PDK1 reprograms breast cancer stem cells under hypoxia. Oncogene. (2018) 37:1062–74. 10.1038/onc.2017.368 29106390 PMC5851116

[20] Du L Yang Y Xiao X Wang C Zhang X Wang L. Sox2 nuclear expression is closely associated with poor prognosis in patients with histologically node-negative oral tongue squamous cell carcinoma. Oral Oncol. (2011) 47:709–13. 10.1016/j.oraloncology.2011.05.017 21689966

[21] Ma L Yao Z Deng W Zhang D Zhang H. The many faces of MTA3 protein in normal development and cancers. Curr Prot Peptide Sci. (2016) 17:726–34. 10.2174/1389203717666160401150122 27033852

[22] Li X Jia S Wang S Wang Y Meng A. Mta3-NuRD complex is a master regulator for initiation of primitive hematopoiesis in vertebrate embryos. Blood. (2009) 114:5464–72. 10.1182/blood-2009-06-227777 19864643

[23] Si W Huang W Zheng Y Yang Y Liu X Shan L. Dysfunction of the reciprocal feedback loop between GATA3- and ZEB2-nucleated repression programs contributes to breast cancer metastasis. Cancer Cell. (2015) 27:822–36. 10.1016/j.ccell.2015.04.011 26028330

[24] Du L Ning Z Zhang H Liu F. Corepressor metastasis-associated protein 3 modulates epithelial-to-mesenchymal transition and metastasis. Chin J Cancer. (2017) 36:28. 10.1186/s40880-017-0193-8 28279208 PMC5345190

[25] Fujita N Jaye DL Kajita M Geigerman C Moreno CS Wade PA. MTA3, a Mi-2/NuRD complex subunit, regulates an invasive growth pathway in breast cancer. Cell. (2003) 113:207–19. 10.1016/S0092-8674(03)00234-4 12705869

[26] Zhang H Stephens LC Kumar R. Metastasis tumor antigen family proteins during breast cancer progression and metastasis in a reliable mouse model for human breast cancer. Clin Cancer Res. (2006) 12:1479–86. 10.1158/1078-0432.CCR-05-1519 16533771

[27] Dong H Guo H Xie L Wang G Zhong X Khoury T. The metastasis-associated gene MTA3, a component of the Mi-2/NuRD transcriptional repression complex, predicts prognosis of gastroesophageal junction adenocarcinoma. PLoS ONE. (2013) 8:e62986. 10.1371/journal.pone.0062986 23671646 PMC3643958

[28] Shan S Hui G Hou F Shi H Zhou G Yan H. Expression of metastasis-associated protein 3 in human brain glioma related to tumor prognosis. Neurol Sci. (2015) 36:1799–804. 10.1007/s10072-015-2252-8 26002011

[29] Zhang H Singh RR Talukder AH Kumar R. Metastatic tumor antigen 3 is a direct corepressor of the Wnt4 pathway. Genes Dev. (2006) 20:2943–8. 10.1101/gad.1461706 17050676 PMC1620027

[30] Singh RR Kumar R. MTA family of transcriptional metaregulators in mammary gland morphogenesis and breast cancer. J Mammary Gland Biol Neoplasia. (2007) 12:115–25. 10.1007/s10911-007-9043-7 17549610

[31] Mylonas I Bruning A. The metastasis-associated gene MTA3 is an independent prognostic parameter in uterine non-endometrioid carcinomas. Histopathology. (2012) 60:665–70. 10.1111/j.1365-2559.2011.04103.x 22235751

[32] Huang Y Li Y He F Wang S Li Y Ji G. Metastasis-associated protein 3 in colorectal cancer determines tumor recurrence and prognosis. Oncotarget. (2017) 8:37164–71. 10.18632/oncotarget.16332 28418887 PMC5514899

[33] Feng Y Ke C Tang Q Dong H Zheng X Lin W. Metformin promotes autophagy and apoptosis in esophageal squamous cell carcinoma by downregulating Stat3 signaling. Cell Death Dis. (2014) 5:e1088. 10.1038/cddis.2014.59 24577086 PMC3944271

[34] Wang L Li K Lin X Yao Z Wang S Xiong X. Metformin induces human esophageal carcinoma cell pyroptosis by targeting the miR-497/PELP1 axis. Cancer Lett. (2019) 450:22–31. 10.1016/j.canlet.2019.02.014 30771436

[35] Wang L Li W Li K Guo Y Liu D Yao Z. The oncogenic roles of nuclear receptor coactivator 1 in human esophageal carcinoma. Cancer Med. (2018) 7:5205–16. 10.1002/cam4.1786 30270520 PMC6198200

[36] Chen C Mendez E Houck J Fan W Lohavanichbutr P Doody D. Gene expression profiling identifies genes predictive of oral squamous cell carcinoma. Cancer Epidemiol Biomarkers Prev. (2008) 17:2152-2162. 10.1158/1055-9965.EPI-07-2893 18669583 PMC2575803

[37] Peng CH Liao CT Peng SC Chen YJ Cheng AJ Juang JL. A novel molecular signature identified by systems genetics approach predicts prognosis in oral squamous cell carcinoma. PLoS ONE. (2011) 6:e23452. 10.1371/journal.pone.0023452 21853135 PMC3154947

[38] Enokida T Fujii S Takahashi M Higuchi Y Nomura S Wakasugi T. Gene expression profiling to predict recurrence of advanced squamous cell carcinoma of the tongue: discovery and external validation. Oncotarget. (2017) 8:61786–99. 10.18632/oncotarget.18692 28977904 PMC5617464

[39] Rentoft M Coates PJ Laurell G Nylander K. Transcriptional profiling of formalin fixed paraffin embedded tissue: pitfalls and recommendations for identifying biologically relevant changes. PLoS ONE. (2012) 7:e35276. 10.1371/journal.pone.0035276 22530001 PMC3328434

[40] Vidal SJ Rodriguez-Bravo V Galsky M Cordon-Cardo C Domingo-Domenech J. Targeting cancer stem cells to suppress acquired chemotherapy resistance. Oncogene. (2014) 33:4451–63. 10.1038/onc.2013.411 24096485

[41] Vered M Allon I Buchner A Dayan D. Stromal myofibroblasts and malignant transformation in a 4NQO rat tongue carcinogenesis model. Oral Oncol. (2007) 43:999–1006. 10.1016/j.oraloncology.2006.11.007 17257886

[42] Chen YF Yang CC Kao SY Liu CJ Lin SC Chang KW. MicroRNA-211 enhances the oncogenicity of carcinogen-induced oral carcinoma by repressing TCF12 and increasing antioxidant activity. Cancer Res. (2016) 76:4872–86. 10.1158/0008-5472.CAN-15-1664 27221705

[43] Ning Z Gan J Chen C Zhang D Zhang H. Molecular functions and significance of the MTA family in hormone-independent cancer. Cancer Metast. Rev. (2014) 33:901–19. 10.1007/s10555-014-9517-1 25341508

[44] Kumar R. Another tie that binds the MTA family to breast cancer. Cell. (2003) 113:142–3. 10.1016/S0092-8674(03)00274-5 12705862

[45] Li DQ Pakala SB Nair SS Eswaran J Kumar R. Metastasis-associated protein 1/nucleosome remodeling and histone deacetylase complex in cancer. Cancer Res. (2012) 72:387–94. 10.1158/0008-5472.CAN-11-2345 22253283 PMC3261506

[46] Singh SK Chen NM Hessmann E Siveke J Lahmann M Singh G. Antithetical NFATc1-Sox2 and p53-miR200 signaling networks govern pancreatic cancer cell plasticity. EMBO J. (2015) 34:517–30. 10.15252/embj.201489574 25586376 PMC4331005

[47] Mu P Zhang Z Benelli M Karthaus WR Hoover E Chen CC. SOX2 promotes lineage plasticity and antiandrogen resistance in TP53- and RB1-deficient prostate cancer. Science. (2017) 355:84–8. 10.1126/science.aah4307 28059768 PMC5247742

[48] Tan Y Tajik A Chen J Jia Q Chowdhury F Wang L. Matrix softness regulates plasticity of tumour-repopulating cells via H3K9 demethylation and Sox2 expression. Nat Commun. (2014) 5:4619. 10.1038/ncomms5619 25099074 PMC4133791

[49] Maurizi G Verma N Gadi A Mansukhani A Basilico C. Sox2 is required for tumor development and cancer cell proliferation in osteosarcoma. Oncogene. (2018) 37:4626–32. 10.1038/s41388-018-0292-2 29743593 PMC6195857

[50] Wang K Ji W Yu Y Li Z Niu X Xia W. FGFR1-ERK1/2-SOX2 axis promotes cell proliferation, epithelial-mesenchymal transition, and metastasis in FGFR1-amplified lung cancer. Oncogene. (2018) 37:5340–54. 10.1038/s41388-018-0311-3 29858603

[51] Liu X Qiao B Zhao T Hu F Lam AK Tao Q. Sox2 promotes tumor aggressiveness and epithelialmesenchymal transition in tongue squamous cell carcinoma. Int J Mol Med. (2018) 42:1418–26. 10.3892/ijmm.2018.3742 29956740 PMC6089783

