# Supplementary material for: MTA3-SOX2 Module Regulates Cancer Stemness and Contributes to Clinical Outcomes of Tongue Carcinoma
Source: Front Oncol. 2019 Aug 27;9:816. doi: 10.3389/fonc.2019.00816 (PMC6736560; doi:10.3389/fonc.2019.00816)
Supplement: Supplementary file 1 [file Data_Sheet_1.PDF]

Supplementary Material

Supplementary Figures

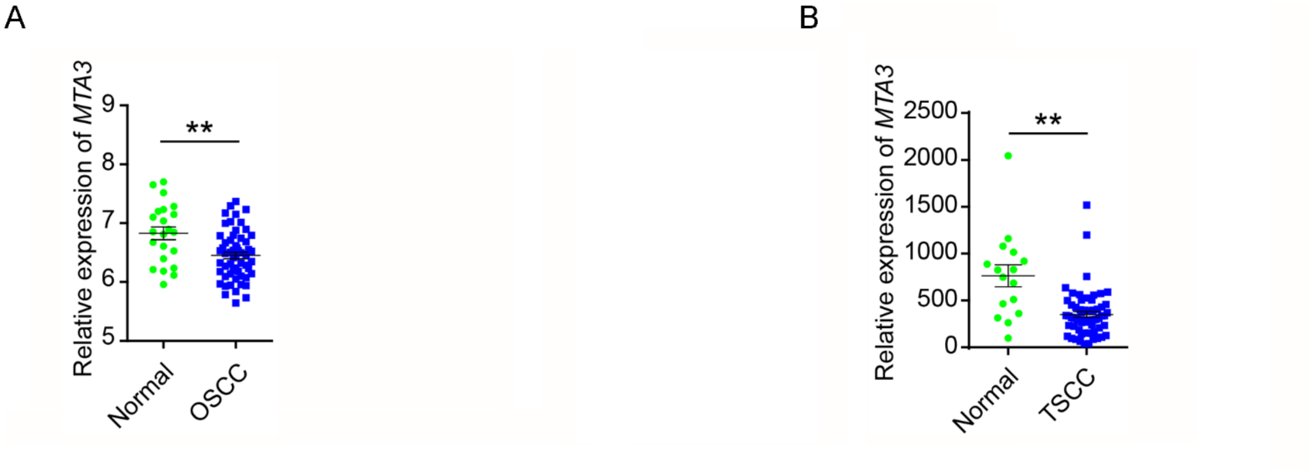

Supplementary Figure 1. MTA3 mRNA is downregulated in human TSCC. (**A**) The mRNA levels of MTA3 in an OSCC dataset from GEO (GSE25099) was compared to that of the normal control. (**B**) The mRNA levels of MTA3 in a published TSCC dataset from GEO (GSE34105) was performed with that of normal control. Error bars indicate SEM. $^{**}p < 0.01$ by Student's *t* test.

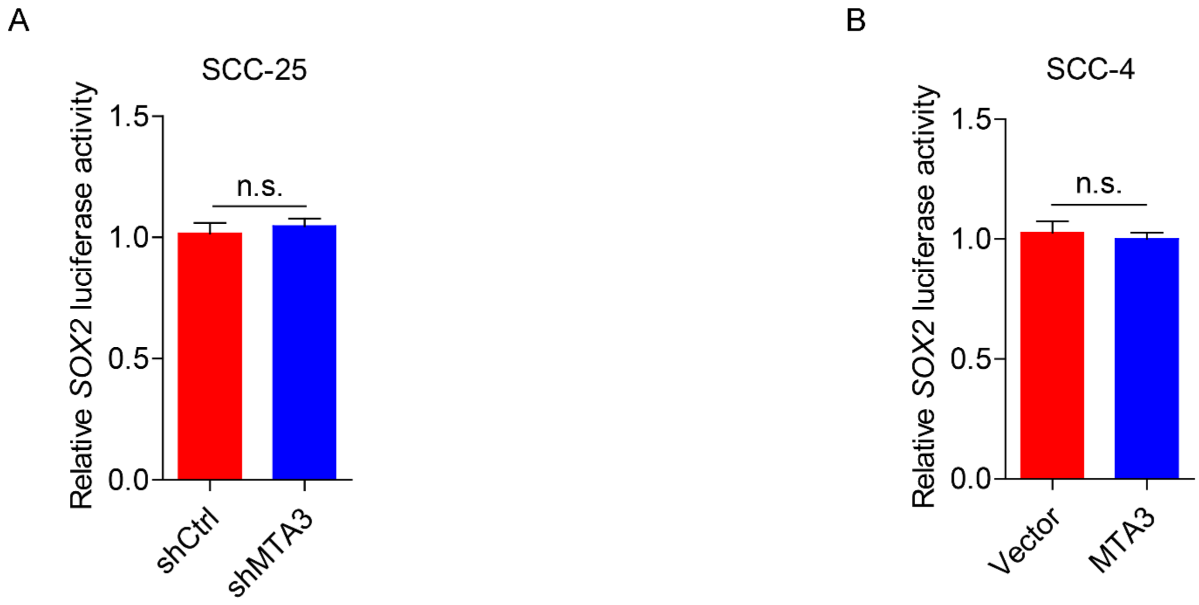

Supplementary Figure 2. MTA3 does not regulate the expression of SOX2 by interacting with its proximal promoter. (**A** and **B**) SOX2 luciferase reporter was transfected into TSCC cells with MTA3 depleted (A) or overexpressed (B). The relative SOX2 luciferase reporter activities were measured at 72 h after transfection. Data were shown as the means from at least three independent experiments or representative data. Error bars indicate Error bars indicate SEM. n.s., not statistically significant by Student's t test.
